# Immediate Mood Scaler: Tracking Symptoms of Depression and Anxiety Using a Novel Mobile Mood Scale

**DOI:** 10.2196/mhealth.6544

**Published:** 2017-04-12

**Authors:** Mor Nahum, Thomas M Van Vleet, Vikaas S Sohal, Julie J Mirzabekov, Vikram R Rao, Deanna L Wallace, Morgan B Lee, Heather Dawes, Alit Stark-Inbar, Joshua Thomas Jordan, Bruno Biagianti, Michael Merzenich, Edward F Chang

**Affiliations:** ^1^ School of OT, Faculty of Medicine Hebrew University Jerusalem Israel; ^2^ Posit Science Corporation San Francisco, CA United States; ^3^ Department of Psychiatry University of California San Francisco, CA United States; ^4^ UC Berkeley- UCSF Joint Medical Program University of California Berkeley, CA United States; ^5^ School of Medicine University of California San Francisco, CA United States; ^6^ Department of Neurology University of California San Francisco, CA United States; ^7^ Department of Neurosurgery University of California San Francisco, CA United States; ^8^ Department of Psychology University of California Berkeley, CA United States; ^9^ California School of Professional Psychology Alliant International University San Francisco, CA United States

**Keywords:** mood disorders, mobile, ecological momentary assessment, depression, anxiety

## Abstract

**Background:**

Mood disorders are dynamic disorders characterized by multimodal symptoms. Clinical assessment of symptoms is currently limited to relatively sparse, routine clinic visits, requiring retrospective recollection of symptoms present in the weeks preceding the visit. Novel advances in mobile tools now support ecological momentary assessment of mood, conducted frequently using mobile devices, outside the clinical setting. Such mood assessment may help circumvent problems associated with infrequent reporting and better characterize the dynamic presentation of mood symptoms, informing the delivery of novel treatment options.

**Objectives:**

The aim of our study was to validate the Immediate Mood Scaler (IMS), a newly developed, iPad-deliverable 22-item self-report tool designed to capture current mood states.

**Methods:**

A total of 110 individuals completed standardized questionnaires (Patient Health Questionnaire, 9-item [PHQ-9]; generalized anxiety disorder, 7-Item [GAD-7]; and rumination scale) and IMS at baseline. Of the total, 56 completed at least one additional session of IMS, and 17 completed one additional administration of PHQ-9 and GAD-7. We conducted exploratory Principal Axis Factor Analysis to assess dimensionality of IMS, and computed zero-order correlations to investigate associations between IMS and standardized scales. Linear Mixed Model (LMM) was used to assess IMS stability across time and to test predictability of PHQ-9 and GAD-7 score by IMS.

**Results:**

Strong correlations were found between standard mood scales and the IMS at baseline (*r*=.57-.59, *P*<.001). A factor analysis revealed a 12-item IMS (“IMS-12”) with two factors: a “depression” factor and an “anxiety” factor. IMS-12 depression subscale was more strongly correlated with PHQ-9 than with GAD-7 (*z*=1.88, *P*=.03), but the reverse pattern was not found for IMS-12 anxiety subscale. IMS-12 showed less stability over time compared with PHQ-9 and GAD-7 (.65 vs .91), potentially reflecting more sensitivity to mood dynamics. In addition, IMS-12 ratings indicated that individuals with mild to moderate depression had greater mood fluctuations compared with individuals with severe depression (.42 vs .79; *P*=.04). Finally, IMS-12 significantly contributed to the prediction of subsequent PHQ-9 (beta=1.03, *P*=.02) and GAD-7 scores (beta =.93, *P*=.01).

**Conclusions:**

Collectively, these data suggest that the 12-item IMS (IMS-12) is a valid tool to assess momentary mood symptoms related to anxiety and depression. Although IMS-12 shows good correlation with standardized scales, it further captures mood fluctuations better and significantly adds to the prediction of the scales. Results are discussed in the context of providing continuous symptom quantification that may inform novel treatment options and support personalized treatment plans.

## Introduction

Mood disorders such as anxiety and depression afflict a significant portion of the population and pose a huge burden in total disability-adjusted years among midlife adults [1-5]. Mood disorders are often dynamic disorders, with symptoms showing high interpatient variability, as well as high intrapatient changes over time. However, our ability to accurately characterize day-to-day variation in these symptoms is limited by current standard of care, which is composed primarily of retrospective self-reports and subjective clinical impression, often during infrequent clinical visits [6-10]. Thus, despite their clinical significance, most symptoms are not continuously tracked outside the clinical setting or between treatment sessions [11].

Monitoring patients more frequently outside of the clinical setting, in “the real world” may improve clinical care and help facilitate timely interventions. First, capturing the relationship between mood dynamics and disease profile may pave the way for a better understanding and classification of disease and allow for improved accuracy of diagnosis and personalization of treatment [12,13]. Several recent studies have shown the clinical significance of temporal fluctuations in mood symptoms, noting the dynamic nature of mood characteristics that often go unreported, and the lost potential to better guide treatment planning [9,14-17]. Specifically, variations in positive and negative affect have been linked to the current level of depression, and increased variability in mood ratings predicted future depressive episodes [18-22]. However, there is an ongoing debate as to how mood fluctuations and variability in mood symptoms over time are associated with the severity of disease at onset (see [21] for a recent review), which may be resolved by data collected through consistent mood tracking that should provide better disease classification and ultimately improved personalized diagnosis and treatment.

Second, mobile mood tracking may help eliminate the potential reporting bias which arises when patients are required to retrospectively recall and rate symptoms, often of a distressing nature, that occurred over the past weeks or months [23,24]. Such mood reporting, particularly among those experiencing mood disruptions, is known to be associated with a large number of recall biases and erroneous judgments [25-29], such as “reconstruction” of memories [30,31] or excessive reliance on cognitive heuristics [32,33]. It has further been shown that mood reporting at the time of recall can also bias memory, making mood-congruent information more prone to be reported [34]. Finally, individuals suffering from mood disorder have been shown to have cognitive limitations, such as working memory deficits, which may obscure the utility of such reporting [35-41].

Third, identification of environmental factors relevant to mood symptoms and intervention can lead to personalized and more effective care. In addition to inaccurately captured mood fluctuations and potential biases, standard assessment in the clinic, rather than in the individual’s natural ecologically relevant settings, is likely to significantly limit the ability to assess true mood state. Assessing a person’s mood in their everyday settings, with further understanding of typical scenarios that influence mood state, may provide better and more complete avenues for treatment, more easily incorporated into day-to-day activities. The fact that more than 75% of patients suffer a depressive episode again within 2 years of treatment [42], which has been partly accounted for by poor continuity of care, further necessitates immediate mood tracking, performed under more ecologically valid conditions and outside of standard care.

Recent advances in mobile “smart” technologies may now facilitate remote tracking and monitoring of patients with mood disorders in their natural environment, and may thus help overcome barriers to treatment success and reporting biases, and ensure better continuity of care [8-10,14,43-45]. As patients with mood disorders are increasingly using mobile technology [10], mobile mood apps offer a convenient ecological momentary assessment mechanism to capture patients’ status in real time [8]. Approaches to ecological momentary mood assessment in psychiatric patients have received some research support in studies showing feasibility of use in depression screening using a mobile phone app [7,12,13,17,46], and in patients’ capability to fill out questionnaires for quantitative data entry [6,47,48]. Similar results were reported by Torous et al [49], who used a mobile phone app to administer a subset of PHQ-9 questions to capture depressive symptoms in psychiatric outpatients. Others [16,50,51] have also examined the feasibility of daily or weekly short message service (SMS)–based mood ratings and found these ratings to be a valid monitoring strategy for depressed participants. Such studies provide initial promising evidence for the utility of remote momentary assessments, and additional evidence is required in order to better establish the usability of such tools. Notably, although data from some of these studies suggest that daily mood reporting may provide more accurate indicator of longitudinal symptoms [16,47], further understanding of the nature of mood fluctuations captured on a mobile device in ecologically valid setting is necessary and would potentially provide a powerful tool to inform treatment in patients with mood disorders.

This study was designed to assess the utility of a novel mobile mood tracking scale, the Immediate Mood Scaler (IMS), a quick 22-item scale which asks participants to rate mood-related constructs in the moment. IMS was delivered along with standardized mood-related questionnaires within a single mobile app (the Mobile Mood Tracker), thus allowing us to evaluate its efficacy for accurately characterizing the current level of depression or anxiety (ie, mood) outside the clinic. We further aimed to assess the dynamic range of mood ratings over time, and test the hypothesis that the variability of mood ratings provides additional information in predicting levels of depression and anxiety [52].

## Methods

### Recruitment and Enrollment

A convenience sample of 110 participants was included in the study and completed the assessments using iPads (see details below). Participants were recruited from three sites: 75 participants were patients at the Epilepsy Monitoring Unit (EMU) of the University of California, San Francisco (UCSF) Medical Center, 24 participants were recruited through the University of California, Berkeley (UCB) Department of Psychology, and 11 participants were recruited through Posit Science (PSC).

Participants at UCSF EMU were recruited as part of broader efforts to examine daily mood fluctuations, while participants were hospitalized for seizure monitoring and probing neural correlates with electroencephalography (EEG) and electrocorticography (ECoG) [53]. These participants were enrolled in the study during their stay at the EMU. UCB participants were recruited through the Research Participant Pool and received course credit for completing the study. PSC participants were recruited through Web-based ads. UCSF EMU patients were consented for research studies, including mood assessment with the app, on a study-provided mini iPad. UCB and PSC participants gave written informed consent before using the app. The study was run under the institutional review board (IRB) approvals from UCSF, UCB, and Western IRB. Participants were not paid for their participation in the study.

Note that although we have two separate subgroups in our sample (considering the PSC and UCB samples to be similar), and that we estimated that they would be quantitatively independent groups per intraclass correlations (ICC), random coefficient models suggested that the cohorts did not observe different associations between variables. Thus, because the correlations between variables were similar across groups, we decided to treat the group as one sample in the analysis.

### Study Procedures

**Figure 1:**
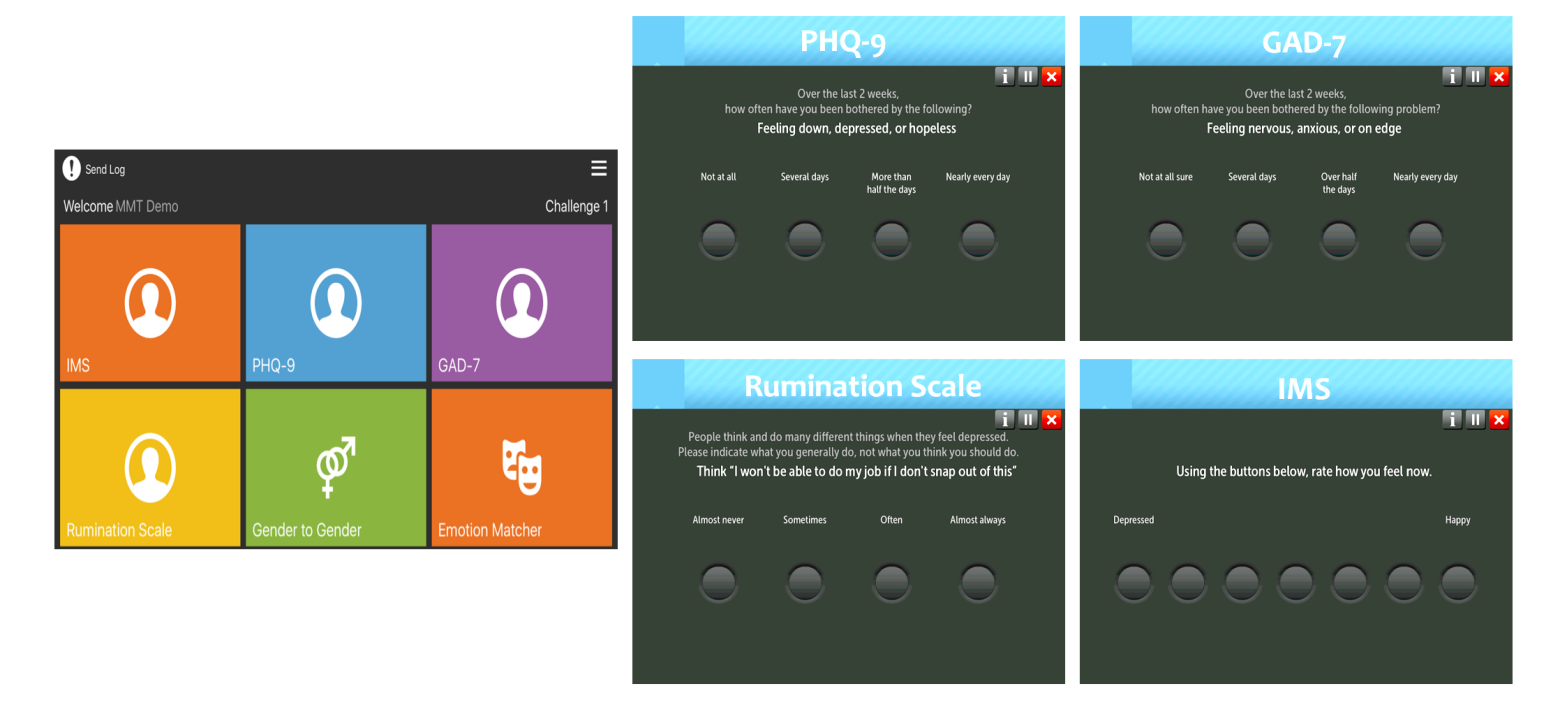
Posit Science’s mobile mood tracker app. Left: the app’s intro screen on the iPad. The user clicks on any tile to start the assessment. Right: single example items from PHQ-9, GAD-7, Rumination, and IMS are shown. PHQ-9: patient health questionnaire, 9-item. GAD-7: generalized anxiety disorder, 7-item. IMS: Immediate Mood Scaler.

Following informed consent, participants were given an iPad mini (Model # A1454, iPad mini WiFi 16GB; Apple, Inc) and were asked to log in to PSC’s Mobile Mood Tracker app with a unique password-protected login to complete the tasks (Figure 1). UCSF EMU participants completed the procedure during their hospitalization (in clinic) and UCB or PSC participants completed it in the lab at UC Berkeley or at the PSC offices in San Francisco. Data were saved on a password-protected Health Insurance Portability and Accountability Act (HIPAA)–compliant server, accessible to study investigators only through a Web browser. Study participants completed at least one session (with a variable number of assessments completed, see below). To obtain repeated-use data, 56 of the participants (all EMU patients) agreed to repeat IMS administration at least one more time. Of them, 17 participants also repeated the PHQ-9 and GAD-7 questionnaires a second time. Below is the list of mobile assessments completed by study participants:

#### Immediate Mood Scale (IMS)

A novel 22-item measure developed to assess dynamic components of mood. Participants were asked to rate their current mood state on a continuum using 7-point Likert scales (eg, happy-sad, distracted-focused, sleepy-alert, fearful-fearless. For each item, an integer score between 1 and 7 was derived. The total score for this scale is the sum of the scores on all 22 items. To make this scale in-line with the scores derived from the PHQ-9 measure, we then inverted the total score received, such that higher scores reflect more negative mood states. Baseline IMS data were obtained from all 110 participants. A complete list of the 22 IMS items can be found in Multimedia Appendix 1 and a video demo of the IMS can be found in Multimedia Appendix 2.

#### Patient Health Questionnaire, 9-Item (PHQ-9)

A standardized, validated 9-item self-report questionnaire used to assess DSM-V-TR [55] symptoms of depression experienced in the 2 weeks preceding administration in adults [54]. We used this measure to classify participants into the following categories, per PHQ-9 cut-off scores (total scores range from 0 to 27): minimal depression (0-4), mild depression (5-9), moderate depression (10-14), moderately severe depression (15-19), and severe depression (20 and over). Baseline PHQ-9 data were obtained from all the 110 participants. PHQ-9 is the most commonly administered self-report tool for depression, has good diagnostic and psychometric properties, and has been shown to be valid across numerous modes of administration [56].

#### Generalized Anxiety Disorder, 7-Item (GAD-7)

A standardized, validated 7-item self-report questionnaire used to assess symptoms of anxiety experienced in the 2 weeks preceding administration [57]. We used this measure to classify participants in the following categories, per GAD-7 cut-off scores (total scores range from 0 to 21): minimal anxiety (0-4), mild anxiety (5-9), moderate anxiety (10-14), and severe anxiety (15 and over). Baseline GAD-7 data were obtained from 93 of the 110 participants (84.5%) since this scale was added at a later stage. GAD-7 has good reliability and validity metrics.

#### Ruminative Responses Scale (Rumination)

A standardized, validated 22-item self-report questionnaire used to assess level of rumination experienced in the 2 weeks preceding administration [58]. As this scale was added to app at a later stage of the study, baseline rumination data were obtained from only 64 of the 110 participants (58.1%).

### Data Analysis

All statistical analyses were conducted using Stata (StataCorp LP). Sample demographics (age and gender) were analyzed using descriptive statistics, and were compared using independent sample *t* tests with the Welch-Satterthwaite correction (age) and with Pearson chi-square test (gender).

To examine relationship between IMS and standard scales (PHQ-9, GAD-7, rumination) at baseline, we computed zero-order correlations using Pearson *r* to investigate possible associations between PHQ-9, GAD-7, rumination, and IMS. The difference between correlations was examined using the test for comparing elements of a correlation matrix [59], using a Web-based tool [60].

To perform dimensionality reduction and factor analysis of IMS, we conducted an exploratory principal axis factor analysis with Promax rotation on all items comprising the IMS, with the global item removed. We used parallel analysis [61] with 1000 simulations of the raw data to identify the number of factors to retain, and considered factors present if they exceeded the simulated eigenvalue. Internal consistency of the solution was tested using Cronbach’s alpha.

To test stability of the total IMS score and subscales across time (repeated observations), we used a linear mixed model (LMM [62]) which allows for repeated observations and tolerates missing data, a common occurrence in repeated-measures designs. Stability was estimated using ICC.

Finally, to test predictability of PHQ-9 and GAD-7 scores by IMS, we conducted an exploratory analysis on the subset of participants that had multiple data points for these scales using LMMs. Due to the small sample size, we used restricted maximum likelihood estimation and applied Satterthwaite degrees of freedom to provide a more conservative test of significance. Predictors in these models were standardized before analysis to facilitate interpretation of the coefficients.

## Results

### Characterization of Study Sample

**Figure 2:**
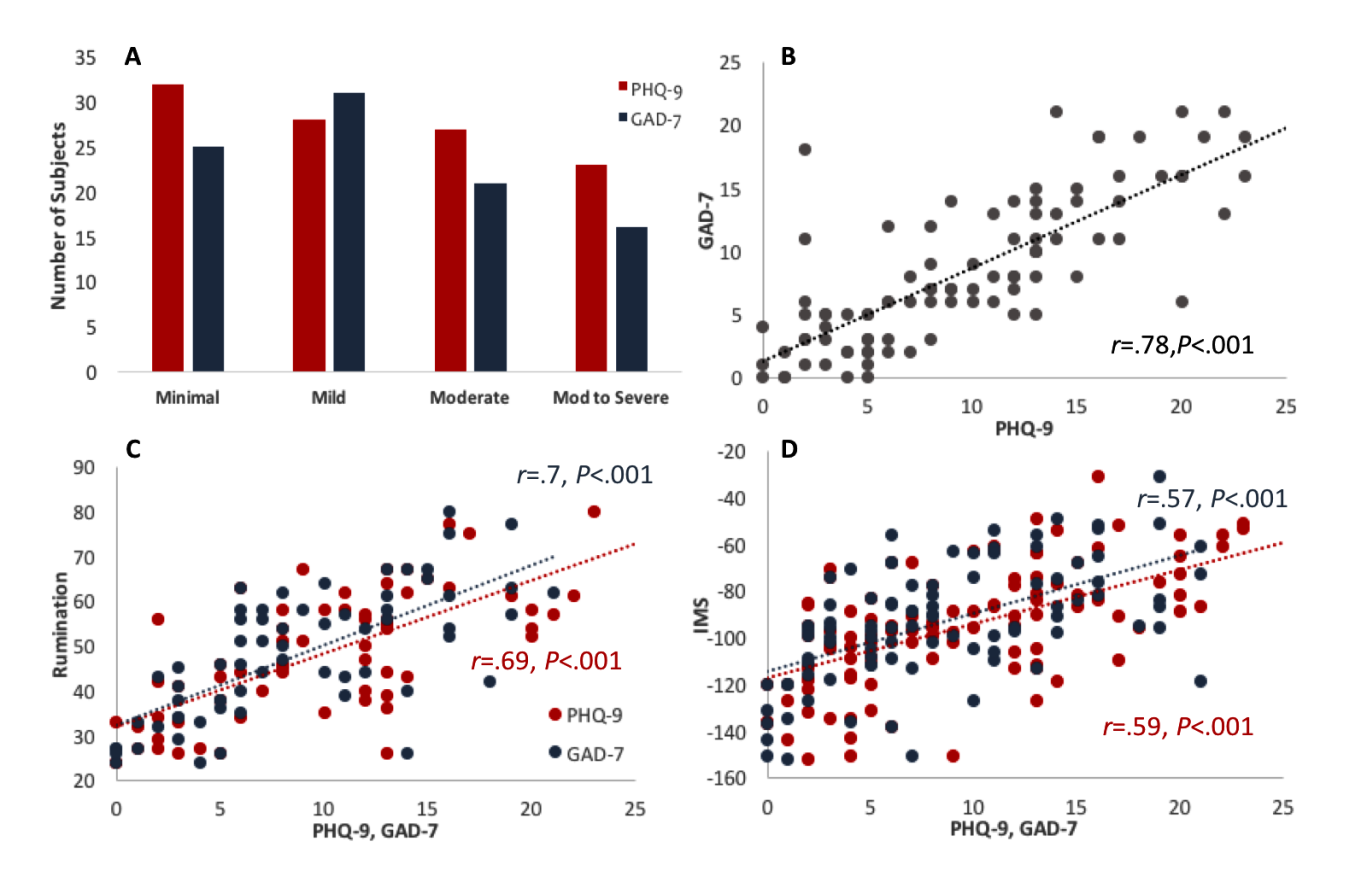
(A) PHQ-9 (red bars) and GAD-7 (blue bars) score distribution. Since the GAD-7 scale only has 4 categories and PHQ-9 has 5 categories, we have included PHQ-9 scores of moderately severe to severe in the “Mod to Severe” category. (B) PHQ-9 individual score correlation with the GAD-7 scale. (C) Correlation between PHQ-9 (red) and GAD-7 (blue) scales and the rumination scale. (D) Correlation between PHQ-9 (red) and GAD-7 (blue) scores with the full IMS score. PHQ-9: patient health questionnaire, 9-item. GAD-7: generalized anxiety disorder, 7-item. IMS: Immediate Mood Scaler.

Participants’ age range was 18-63 years old (average: 34 years, SD 11.8). Of the 110 participants, 64 (58.1%) were female, 32 were classified as having minimal or no depression (PHQ-9 scores of 0-4; mean age 31, SD 11.8), 28 with mild depression (PHQ-9 scores of 5-9; mean age 30, SD 8.3), 27 with moderate depression (PHQ-9 scores of 10-14; mean age 37.3, SD 13.5), 12 with moderately severe depression (PHQ-9 scores of 15-19; mean age 36.2, SD 10.6), and 11 with severe depression (PHQ-9 scores of 20-27; mean age 46.8, SD 6.9). The study sample is depicted in Multimedia Appendix 3 and in Figure 2.

Comparing the two participant groups in our sample (UCSF, and UCB or PSC samples) showed that the two groups differed significantly in age (*t*_54_=4.3, *P*<.001), but not in PHQ-9 (*t*_67_=1.8, *P*=.07) or in gender (χ^2^_2_=.5, *P*=.76).

### Correlation Between Measures at Baseline

We examined the correlation between PHQ-9, GAD-7, and rumination scale, as well as the correlation between these scales and IMS. As expected, the standardized depression scale (PHQ-9) was highly correlated with the standardized anxiety scale (GAD-7; *r*=.78, *P*<.001; Figure 2), pointing to the frequent comorbidity of depression and anxiety. Furthermore, the PHQ-9 and GAD-7 were both highly correlated with the rumination scale (*r*=.69 and .70, *P*<.001 for PHQ-9 and GAD-7, respectively; Figure 2). The IMS total score was highly correlated with PHQ-9 (*r*=.59, *P*<.001; n=110) and with GAD-7 (*r*=.57, *P*<.001; n=93) scales (Figure 2), as well as with rumination scale (*r*=.57, *P*<.001; n=64; data not shown).

### Dimensionality Reduction and Factor Analysis for the Immediate Mood Scaler (IMS)

To assess factorial validity and to identify which items needed to be removed from the IMS to provide briefer assessment, we conducted an exploratory principal axis factor analysis. Although our sample size was not ideal for a factor analysis (N=110), the Kaiser-Meyer-Olkin (KMO) [63] measure of sampling adequacy (.91) and Bartlett Test of Sphericity [64] (χ^2^_231_=1560.35, *P*<.001) indicated that a factor analysis was appropriate for the data. We first identified the number of factors to retain through parallel analysis [61] on the raw data with 1000 simulations. A factor was considered present if it exceeded the simulated eigenvalue. This procedure resulted in three underlying factors, which were applied to the data (see Multimedia Appendix 4). Due to the high comorbidity between anxiety and depression, we used an oblique (Promax) rotation to allow the factors to correlate. Because our goal was to first reduce the number of items in the IMS, we examined the pattern matrix and removed items with low loadings (<.40) or items that loaded on more than one factor. We then subjected the remaining 16 items to the same process as outlined above. This resulted in the same 3-factor solution with a depression subscale, an anxiety subscale, and another, weaker 3-item subscale (q5, q6, and q7) which represented energy level. Because our aim was to identify a brief but reliable instrument, we removed the 3-item energy subscale. This resulted in a clear 2-factor solution with excellent internal consistency for the total scale (Cronbach’s alpha=.93) and for the subscales (Cronbach’s alpha=.90 and .93 for depression and anxiety, respectively). This brief 12-item measure (IMS-12) has a near-perfect correlation with the full 22-item IMS scale (*r*=.97, *P*<.001), indicating inconsequential information loss.

The IMS-12 factor analysis results are summarized in Table 1. Items q3, q8, q9, q10, q11, q12, and q16 load between .64 and .83 on factor 1, which seems to capture depressive states (eg, apathetic vs motivated, pessimistic vs optimistic). Items q18-q22 load between .73 and .84 on factor 2, which captures anxiety (eg, worried vs untroubled, anxious vs peaceful).

Following this exploratory analysis, we derived 3 metrics: (1) IMS-12 total score (the sum of the 12 IMS items), (2) IMS-12 depression subscale (a sum of the items loading on factor 1), and (3) an IMS-12 anxiety subscale (a sum of the items loading on factor 2).

**Table 1:** Factor analysis pattern matrix for IMS-12 items.

IMS item			Factor 1 Depression	Factor 2 Anxiety
q3	Worthless	Valuable	*.64* ^a^	.13
q8	Pessimistic	Optimistic	*.69*	−.06
q9	Apathetic	Motivated	*.75*	.20
q10	Guilty	Proud	*.69*	.24
q11	Numb	Interested	*.83*	−.01
q12	Withdrawn	Welcoming	*.71*	.16
q16	Hopeless	Hopeful	*.71*	−.15
q18	Tense	Relaxed	.03	*.83*
q19	Worried	Untroubled	.03	*.83*
q20	Fearful	Fearless	.17	*.73*
q21	Anxious	Peaceful	.09	*.84*
q22	Restless	Calm	.01	*.81*

^a^Values are denoted in italics for the factor they loaded more for.

**Figure 3:**
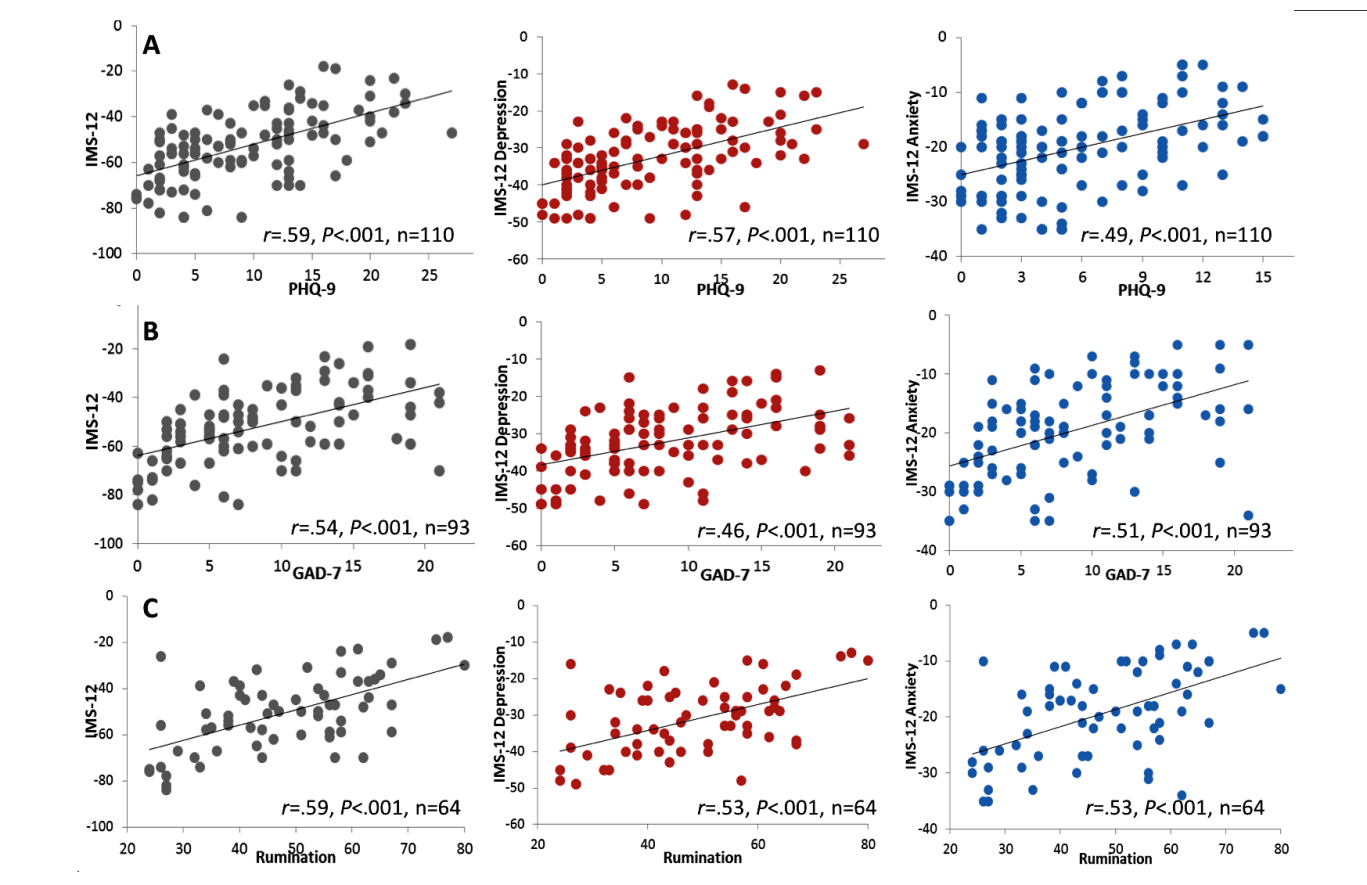
Correlations between IMS-12 and standardized scales. Correlations between IMS-12 total (left, gray), IMS-12 depression (middle, red), and IMS-12 anxiety (right, blue) with PHQ-9 (A; top row), GAD-7 (B; middle row), and rumination (C; bottom row) scales. Pearson r values and number of subjects are shown for each graph. PHQ-9: patient health questionnaire, 9-item. GAD-7: generalized anxiety disorder, 7-item. IMS: Immediate Mood Scaler.

### Relation Between Baseline IMS-12 and Baseline Levels of Depression and Anxiety

We next examined whether IMS subscales were correlated with the PHQ-9 and GAD-7 scales. Correlation results are shown in Figure 3 and in Multimedia Appendix 5. The IMS-12, similarly to the full 22-item scale, was highly correlated with PHQ-9 (*r*=.59, n=110, *P*<.001) and GAD-7 (*r*=.54, n=93, *P*<.001) and rumination (*r*=.59, n=64, *P*<.001) scales, proving that the same correlation is maintained even with a scale featuring a subset of the items (left panels of Figure 3). Of note, we found similar correlations between IMS-16 (with 3 factors) and PHQ-9 and GAD-7 (data not shown).

Similarly, strong correlations were found for IMS-12 depression subscale, which was highly correlated with the PHQ-9 (*r*=.57, n=110, *P*<.001), with GAD-7 (*r*=.46, n=93, *P*<.001) and with rumination (*r*=.53, n=64, *P*<.001). Similarly, the IMS-12 anxiety subscale was highly correlated with PHQ-9 (*r*=.49, n=110, *P*<.001), GAD-7 (*r*=.51, n=93, *P*<.001) and with rumination (*r*=.53, n=64, *P*<.001). Because we hypothesized that IMS-12 depression would have a stronger correlation with the PHQ-9 than with the GAD-7, we tested for the difference in correlations using a one-tailed test of significance. Indeed, the correlation between IMS-12 depression and PHQ-9 was stronger than that of IMS-12 depression and GAD-7 (*z*=1.88, *P*=.03; using Steiger test). However, the correlation between IMS-12 anxiety and GAD-7 was as strong as the correlation between IMS-12 and PHQ-9.

### Time to Administer Scales

Given our goals of producing an efficient measure of mood, we calculated the average time required to complete each of the assessments. On average, it took participants 12.65 s (SD 8) to complete a PHQ-9 item, 8.35 s (SD 4.8) to complete a GAD-7 item and 6.54 s (SD 3.4) to complete an IMS item. An analysis of variance (ANOVA) with Greenhouse-Geisser correction confirmed that the time to complete an IMS item was significantly shorter than the other scales (n=107; *F*_1.29,137_=77.7, *P*<.001).

We further derived the average time it should take to complete the entire scale: PHQ-9 takes, on average, 113.9 s (SD 73.7) to complete, GAD-7 takes 59.5 s (SD 33.5), and IMS-12 takes, on average, 78.4 s (SD 63.8) to complete.

### Analyses of Repeated Administration of IMS

**Figure 4:**
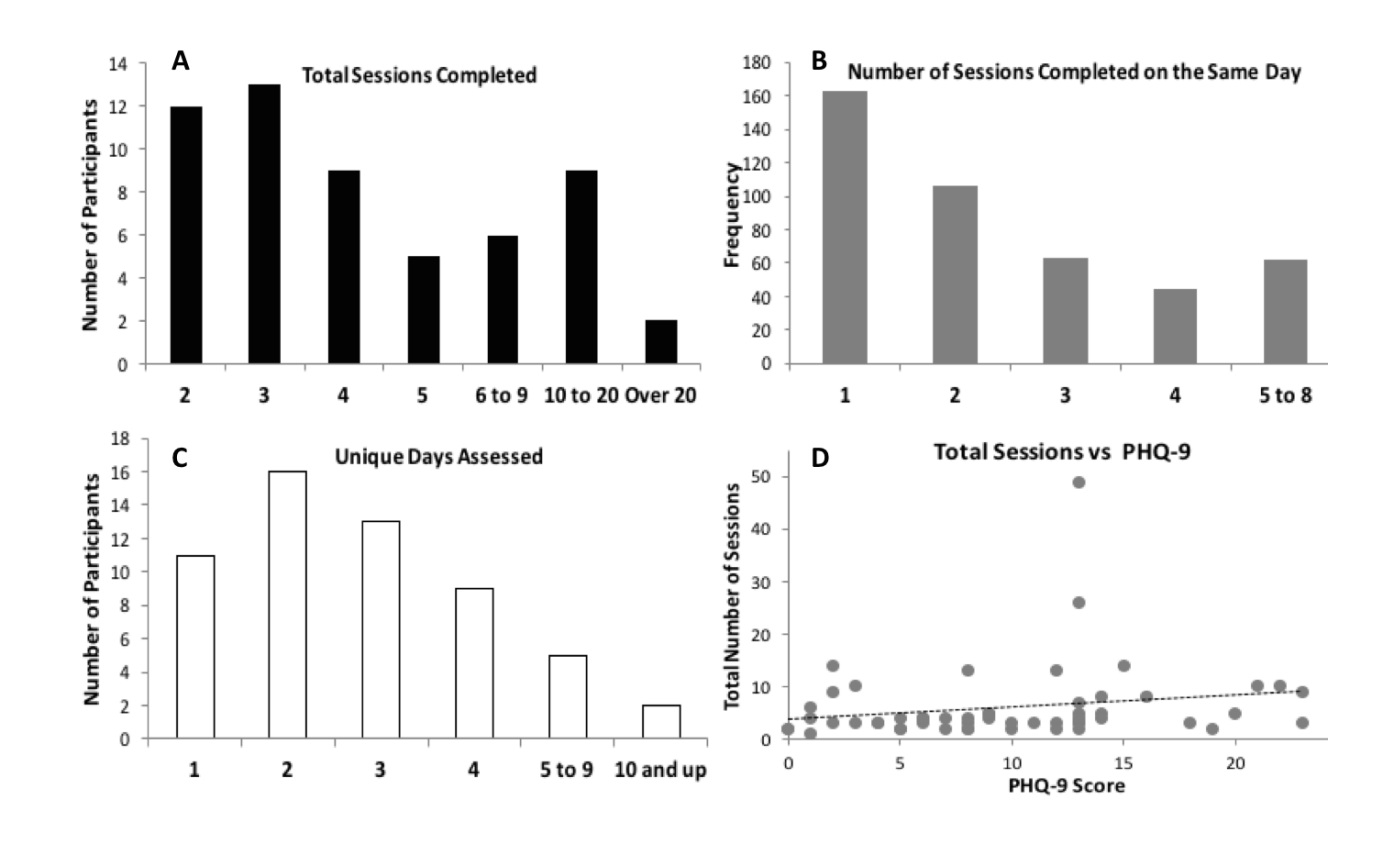
Repeated IMS data frequency. IMS data was collected within days and across days for 56 participants. (A) A histogram showing the total number of sessions completed by participants. (B) Number of sessions completed on the same day (multiple sessions for participants). (C) A histogram showing the unique days of IMS assessments completed by participants. (D) Total number of sessions completed as a function of baseline PHQ-9 score (r=.18, P=.18). PHQ-9: patient health questionnaire, 9-item. IMS: Immediate Mood Scaler.

A total of 56 participants completed two or more sessions during the course of the study, and had IMS data for all repeated sessions they completed. Participants had a variable number of data points, ranging from 2 to 49 (Figure 4), with most participants having 2 or 3 data points of IMS collected (mean 6.5, SD 7.5; median 4). Most data points were collected on the same day, but some were collected on different days (see Figure 4). Number of data points collected did not correlate with severity of symptoms by baseline PHQ-9 (*r*=.18, *P*=.19; Figure 4) or GAD-7 scores (*r*=.01, *P*=.93). Out of those 56 participants, 17 also repeated PHQ-9 and GAD-7 a second time.

### Stability of IMS-12 Scores Across Time and for Different Levels of Depression

We examined the stability of the IMS-12 and its subscales, as well as its ability to predict PHQ-9 and GAD-7 scores administered the second time through the use of LMMs and ICC, taking the maximal number of repeated measures per participant. ICC revealed high test stability for both the PHQ-9 and GAD-7 (ICC=.91 for both) and lower test stability for the IMS-12 (ICC=.65), with similar stability for the depression (ICC=.60) and anxiety (ICC=.61) subscales.

To test the hypothesis that participants with mild to moderate depression levels have greater variability in their mood compared with participants with minimal or severe depression levels, we further examined IMS-12 ICC for different depression levels. Results are summarized in Table 2. Tests for the differences in ICC revealed that individuals with severe depression (PHQ-9 scores of moderately severe to severe) had significantly more consistent mood by the IMS-12 (ie, less fluctuations; ICC=.79) than individuals with mild to moderate depression (ICC=.42; *z*=2.03, *P*=.04). Despite a trend for more consistent mood in individuals with minimal depression than those with mild to moderate, there were no other significant differences between groups; however, this may be due in part to sample size.

**Table 2:** IMS-12 intraclass correlations.

Depression level	N	ICC^a^ (95% CI)	Group comparisons	*z*	*P*
Minimal	39	.69 (.54 to .81)	Minimal versus mild or moderate	1.53	.12
Mild to moderate	27	.42 (.25 to .61)	Minimal versus moderate severe or severe	.78	.43
Moderately severe to severe	21	.79 (.63 to .89)	Mild to moderate vs moderately severe or severe	2.03	.04

^a^ICC: intraclass correlations.

### Predictability of PHQ-9 and GAD-7 by IMS-12

We next asked whether IMS-12 scores predicted PHQ-9 and GAD-7 scores over multiple observations, to determine whether current mood influences self-report ratings of “trait” mood over and above the effects of baseline PHQ-9 and GAD-7.

As noted, there was high test stability in the PHQ-9 and GAD-7, likely a reflection of the instruments’ focus on the previous 2 weeks. Although test stability is high in both measures over the course of repeated observations over several days, we hypothesized that fluctuations in mood may account for some of the variability in PHQ-9 and GAD-7 scores. Because few participants completed the PHQ-9 and GAD-7 more than once (n=17), we conducted an exploratory analysis with that subgroup of participants to predict PHQ-9 and GAD-7 scores over repeated observations via LMMs, in which PHQ-9 and GAD-7 were modeled as a function of time. Due to our small sample size, we used restricted maximum likelihood estimation and applied Satterthwaite degrees of freedom [65] to provide a more conservative test of significance. We examined the incremental effects of the IMS-12 by testing a model that included time and baseline PHQ-9 or GAD-7 as predictors, and included IMS-12 as a time-varying predictor, with subsequent observations (time two and beyond) of PHQ-9 or GAD-7 serving as the dependent variables. Predictors were standardized before analysis to facilitate interpretation of the coefficients.

The results of the model are summarized in Table 3. As can be seen in the table, baseline PHQ-9 scores contributed substantially to the prediction of subsequent PHQ-9 scores, and the addition of IMS-12 to the model significantly predicted PHQ-9 scores beyond baseline PHQ-9 status alone (beta =1.03, *P*=.02). This indicates that the IMS-12 accounts for some of the variability seen in PHQ-9 scores, even when taking into consideration “general” mood. Similar results were seen for GAD-7, with IMS-12 significantly contributing to the prediction of GAD-7 scores, beyond the prediction provided by baseline GAD-7 alone (beta=.91, *P*=.01).

**Table 3:** Model variables for the prediction of PHQ-9 and GAD-7 from time, baseline measurements, and IMS-12.

PHQ-9^a^ (n=17)					GAD-7^b^ (n=17)					
Model	Beta	*t*	Degrees of freedom	*P*	Model	Beta	*t*	Degrees of freedom	*P*
Time	−.13	−0.48	7.79	.65	Time	−.00	−0.01	5.23	.99
+PHQ-9 baseline	3.68	8.83	16.9	<.001	+GAD-7 baseline	4.47	10.45	18.61	<.001
+IMS-12^c^	1.03	2.5	45.47	.02	+IMS-12	.91	2.6	35.98	.01
Intercept	11.05	9.33	1,12.1	<.001	Intercept	9.79	10.94	13.9	<.001

^a^PHQ-9: Patient Health Questionnaire, 9-item.

^b^GAD-7: generalized anxiety disorder, 7-item.

^c^IMS-12: Immediate Mood Scaler, 12-item.

We examined IMS-12 subscales using the same analytic approach, and found that the IMS-12 anxiety subscale significantly predicted PHQ-9 scores (beta=−.97, *t*_85.25_=−2.44, *P*=.02); hhowever, the depression subscale was only near-significant (beta=−.67, *t*_54.19_=−1.84, *P*=.07). For GAD-7, the IMS-12 anxiety subscale significantly predicted GAD-7 scores (beta=−.85, *t*_72.97_=−2.29, *P*=.03), whereas the IMS-12 depression subscale had a similar, albeit only near-significant, effect (beta=−.61, *t*_47.9_=−1.98, *P*=.06). This suggests that IMS-12 anxiety subscale may be a good predictor for both depression and anxiety, whereas the IMS-12 depression subscale does not predict either depression or anxiety to a significant extent. The full model is summarized in Multimedia Appendix 6.

## Discussion

### Principal Findings

The findings of the study provide initial support for the usefulness of the IMS as a tool to remotely and quickly track mood changes related to depression and anxiety in-the-moment. Specifically, we found that a condensed version of IMS comprised of 12 items, IMS-12, is highly correlated with standard scales of depression and anxiety (PHQ-9, GAD-7, and rumination scale). We further found that repeated administration of the IMS-12 provides significant information regarding the participant’s mood state. Specifically, the IMS-12 captured greater variability in mood over time compared with the standard scales of PHQ-9 and GAD-7. Moreover, individuals with moderately severe to severe depression were less variable in IMS-12 over time compared with individuals with mild or moderate depression, indicating greater sensitivity to momentary mood changes especially in the moderate range. Finally, mood fluctuations reflected in repeated IMS-12 administrations significantly accounted for a significant portion of the variability in PHQ-9 and GAD-7 scores, with IMS-12 anxiety subscale better accounting for changes in both PHQ-9 and GAD-7 scores compared with the depression subscale.

### The Use of IMS-12 as a Mobile Mood Tracking Tool

The main goal of our study was to assess the usability of IMS-12 as a novel scale that can be used to assess ecologically valid symptoms related to mood disorders. Collectively, the results of our study support the use of an ecological momentary assessment as a tool to assess fluctuations in symptoms related to mood disorders remotely. Specifically, we found that (1) a novel 12-item scale, IMS-12, shows strong correlation with standard scales of depression and anxiety (PHQ-9, GAD-7, and rumination scale), (2) IMS-12 is comprised of 2 unique factors or subscales (“depression” and “anxiety”), with the IMS-12 depression subscale was found to be more correlated with PHQ-9 scores than the anxiety subscale, and (3) an IMS-12 item is, on average, faster to administer than standard scales.

The results of this study show that IMS-12 can be used as a tool to remotely and quickly track mood and mood state fluctuations over time, both observationally and in response to interventions [66]. Of note, patients also reported, in informal interviews at the end of the study, that the fact that IMS had very little text and only required rating on a continuum made it easier to use than traditional scales, which often include longer text and choices between numbered options. These findings are consistent with several recent reports that have shown good feasibility of similar ecological momentary assessment approaches in patients with mood disorder (eg, major depressive disorder) [47,49,67,68]. Other recent studies further reported good correlation between mobile monitoring tools and standard clinical measures, such as the PHQ-9 measure used in our study [16,50,51]. For example, Aguilera et al [50] found that text messages of daily mood ratings, and their weekly averages (but not their variances or 2 week averages), were highly correlated with paper-and-pencil PHQ-9 scores. They, therefore, suggested that daily assessments of mobile mood ratings may provide a more accurate indicator of longitudinal symptoms, given the recency-bias in the PHQ-9 data. Similar results were obtained by Keding and colleagues [51] and Richmond et al [16], who used a single text message to probe mood and report good correlation with PHQ-9, with even better predictive power.

We further show that the overall IMS-12 total score provides a significant addition to the prediction of both depression (as captured by PHQ-9) and anxiety (as captured via GAD-7). Interestingly, the IMS-12 *anxiety* subscale score had better predictive value for both depression and anxiety than the IMS-12 depression subscale score. These results are in line with those found in a recent study by Keding et al [51]. In their study, the authors found that a single mood item predicted the affective component of PHQ-9, but not its somatic component. The comorbidity of anxiety and depression can sometimes make it challenging to dissociate between the two at the daily reporting level. Indeed, some researchers believe that generalized anxiety should not be considered a disorder of its own, and instead could be considered a marker for the severity of depression [69-71]. However, our results provide support to the notion that the short “anxiety subscale” of IMS-12 may have a good predictive value for both anxiety and depression. The results by Kessler et al [72], providing evidence for the difference in risk factors between anxiety and depression, further support this notion. More research is needed to determine whether anxiety-related symptoms have a better predictive value for mood-related illness progression.

### The Predictive Value of Fluctuations in Mood-Related Symptoms

A secondary aim of the study was to assess the dynamic range of mood ratings over time, and test the hypothesis that the variability of mood ratings provides additional information in predicting levels of depression and anxiety.

Although highly correlated with baseline PHQ-9 and GAD-7 scores, IMS-12 mood ratings were, not surprisingly, less stable over time. Considering that the PHQ-9 and GAD-7 are designed to measure symptoms spanning the previous 2 weeks, whereas the IMS-12 is designed to capture in-the-moment mood status, the lower stability for the IMS-12 and its subscales suggests that the IMS-12 captures fluctuations in mood as expected. Indeed, variability of mood ratings captured in the IMS-12 total score as a function of PHQ-9 baseline scores revealed differences in performance characteristic of the severity of depression. Specifically, individuals with severe depression showed significantly less mood fluctuation compared with those of individuals with mild to moderate depression. This suggests that variability in mood may be used as an index of the severity of depression, and as such, in response to intervention, subsequent greater mood variability in severely depressed individuals may indicate a positive response to treatment.

Interestingly, although recent research suggests that depressed individuals differ from nonclinical populations in the profile of depressed mood during their daily lives [21], there is still an ongoing debate regarding the nature of this difference in relation to fluctuations in mood and mood-related variables (eg, positive and negative affect) [22]. Specifically, although some studies found that individuals with major depressive disorder also show more variable mood states across time [20,68,73-75], others reported “emotional inertia” or less fluctuations in mood over time in more significantly depressed individuals [18,19,52,76-78]. The findings from this study are consistent with an emotional inertia account, that is, more depressed individuals show more preservative pattern of affect [77]. Pemberton and Fuller Tyszkiewicz [21] suggest that the seeming contradiction between stability and variability in mood ratings in depressed individuals could be accounted for by the different time frames used in different studies. Thus, individuals may exhibit both stability in mood (in the short-term) and variability in mood when viewed over a longer time frame. It may be that the mood fluctuations in our study capture the “short-term stability” of mood in severely depressed individuals, and that over longer period of time more fluctuations would be evident. In any event, these fluctuations are informative in characterizing level of depression.

### The Clinical Significance of In-the-Moment, Remote Ecological Mood Monitoring Assessment

The results of this study support similar findings in the recent literature that have shown the significance of remote, in-the-moment (and real-world) approaches to the evaluation of mood state [12,13,17,48]. The feasibility of this approach is supported by the growing usage of mobile devices by patients with mood disorders [9,10,49] and studies that have shown good compliance with mobile monitoring strategies [47,49,79].

Ecological momentary mood assessment has several clear advantages [15,43]. For example, repeated administration of assessments may increase reliability of interpretation and also reduce measurement errors (or misinterpretations). In the case of our app, the fact that IMS-12 scores are more variable than standard mood questionnaires demonstrate its potential to more accurately capture mood fluctuations to better inform treatment planning (eg, quickly determine response to current treatment or potential to benefit from a new treatment, as well as quickly alarm clinicians in case of significant worsening in a patient’s state). IMS-12 can be used to supplement PHQ-9, which has been shown by others to be valid when remotely administered [56], and can be used to assess dynamic processes and changes in mood related to treatments. The fact that PHQ-9 has been shown to reflect a recency effect rather than a 2-week average as it should [50] further stresses the need for a dynamic scale that captures mood “in-the-moment.” Mobile mood tracking tools such as the one used here can therefore help circumvent the retrospective recall bias which is often associated with current methods used by clinicians to assess mood [15,80,81].

The use of a mobile app to report mood has several other potential benefits. For example, the anonymity of reporting mood using an app, rather than informing a clinician or caregiver may provide more accurate mood reporting. This notion received some support from a recent study [49], showing more accurate capture of suicidal ideation in patients using an app compared with in-person reporting. In addition, monitoring data continuously collected using such tools may help inform clinicians about the best treatment option based on the subject’s mood profile, and may further inform the subjects themselves on mood-related behaviors and tendencies as reflected in their continuous monitoring data, that are not readily apparent to them. As more and more data is accumulated that way, significant advances can be made that inform novel therapeutic avenues.

With the rapid development of novel technologies (eg, mobile devices), tracking health-related measures such as mood becomes feasible and accessible to a growing portion of the population. However, in order for it to become standard of care and facilitate clinical work, rigorous testing and validation should take place. However, despite the fact that momentary tracking tools have been around for quite some time, only few have been experimentally tested and even fewer validated [79]. We believe that this initial validation of a mobile scale such as IMS-12 further promotes the likelihood of this approach to aid in clinical care, and further promotes our understanding into illness dynamic manifestation in an ecologically valid manner. Future studies, using mobile phone versions of IMS-12, are needed in order to establish the utility of a mobile mood-tracking platform as a tool that promotes our understanding of the dynamic nature of mood symptoms in everyday lives, and as a tool to monitor and measure treatment response [15,43,82].

### Study Limitations

Our study has several limitations that should be addressed in future research. First, our study sample was a convenience sample, which may have limited generalizability. Second, the sample size with repeated IMS and standardized measures data was small, allowing us to make only exploratory analyses that would need to be confirmed by larger-scale studies. Third, as this study was part of a larger study (with a different research question), we did not collect additional psychiatric data on study participants that may have allowed us to further analyze the data based on participants’ history or clinical profile. Finally, data was collected in the lab and clinic, which may limit its interpretation. Follow-up studies should address these limitations and further establish the value of the IMS-12 as a momentary assessment tool for symptoms related to mood disorders.

## Appendix

Multimedia Appendix 1 A complete list of the Immediate Mood Scaler (IMS) items.

Multimedia Appendix 2 Video demo of the Immediate Mood Scaler (IMS).

Multimedia Appendix 3 Distribution of depression Levels, based on PHQ-9 score at baseline for the entire study population (n=110), for the UCSF participants (n=75), and for the UC Berkeley participants (n=35).

Multimedia Appendix 4 Parallel Analysis based on 1000 simulations of raw data.

Multimedia Appendix 5 Correlations (Pearson r) between standardized measures and IMS. IMS: Immediate Mood Scaler.

Multimedia Appendix 6 Model results using IMS-12 depression and anxiety subscales for depression (PHQ-9; top table) and anxiety (GAD-7; bottom table). IMS: Immediate Mood Scaler. PHQ-9: Patient Health Questionnaire, 9-item.

## Abbreviations

ANOVA: analysis of variance
ECoG: electrocorticography
EEG: electroencephalography
EMU: Epilepsy Monitoring Unit
GAD-7: generalized anxiety disorder, 7-item
HIPAA: Health Insurance Portability and Accountability Act
ICC: intraclass correlations
IMS: Immediate Mood Scaler
IRB: institutional review board
KMO: Kaiser-Meyer-Olkin
PHQ-9: Patient Health Questionnaire, 9-item
PSC: Posit Science
SMS: short message service
UC: University of California
UCB: University of California, Berkeley
UCSF: University of California, San Francisco
