# Supplementary material for: Immediate Mood Scaler: Tracking Symptoms of Depression and Anxiety Using a Novel Mobile Mood Scale
Source: JMIR Mhealth Uhealth. 2017 Apr 12;5(4):e44. doi: 10.2196/mhealth.6544 (PMC5406620; doi:10.2196/mhealth.6544)
Supplement: Multimedia Appendix 1 [file mhealth_v5i4e44_app1.pdf]

Multimedia Appendix 1. A complete list of the Immediate Mood Scaler (IMS) items.

IMS Item		
**q1**	Depressed	Happy
**q2**	Distracted	Focused
**q3**	Worthless	Valuable
**q4**	Lonely	Engaged
**q5**	Sleepy	Alert
**q6**	Slow	Speedy
**q7**	Tired	Energetic
**q8**	Pessimistic	Optimistic
**q9**	Apathetic	Motivated
**q10**	Guilty	Proud
**q11**	Numb	Interested
**q12**	Withdrawn	Welcoming
**q13**	Frustrated	Peaceful
**q14**	Impulsive	Careful
**q15**	Moody	Stable
**q16**	Hopeless	Hopeful
**q17**	Irritable	Easy-going
**q18**	Tense	Relaxed
**q19**	Worried	Untroubled
**q20**	Fearful	Fearless
**q21**	Anxious	Peaceful
**q22**	Restless	Calm
