# Supplementary material for: Immediate Mood Scaler: Tracking Symptoms of Depression and Anxiety Using a Novel Mobile Mood Scale
Source: JMIR Mhealth Uhealth. 2017 Apr 12;5(4):e44. doi: 10.2196/mhealth.6544 (PMC5406620; doi:10.2196/mhealth.6544)
Supplement: Multimedia Appendix 3 [file mhealth_v5i4e44_app3.pdf]

Multimedia Appendix 3. Distribution of depression levels, based on PHQ-9 score at baseline for the entire study population (n=110), for the UCSF/EMU participants (n=75) and for the UC Berkeley/PSC participants (n=35).

Depression Level	PHQ-9 Score Range	# of Subjects	Mean Age (y)	# Female	% Female
**Entire Study Population (n=110)**					
**Minimal**	0-4	32	31±11.8	19	59.4
**Mild**	5-9	28	30±8.3	10	35.7
**Moderate**	10-14	27	37.3±13.5	19	70.4
**Mod-Severe**	15-19	12	36.2±10.6	9	75
**Severe**	20-27	11	46.8±6.9	7	63.6
**TOTAL**		**110**	34.1±11.8	64	**58.2%**
**UCSF EMU Subjects (n=75)**					
**Minimal**	0-4	16	37.1±11.6	10	62.5
**Mild**	5-9	21	30±7	8	38.1
**Moderate**	10-14	22	38.9±12.6	16	72.7
**Mod-Severe**	15-19	8	36.4±6.3	5	62.5
**Severe**	20-27	8	46.8±6.9	6	75
**TOTAL**		**75**	34.1±11.8	45	**60%**
**UCB and PSC Subjects (n=35)**					
**Minimal**	0-4	16	25.3±9.3	9	56.3
**Mild**	5-9	7	30.3±11.5	2	28.6
**Moderate**	10-14	5	30.2±16.7	2	40
**Mod-Severe**	15-19	4	35.8±17.7	4	100
**Severe**	20-27	3	--	1	33.3
**TOTAL**		**35**	27.5±12.1	18	**51.4%**
