# Supplementary figures and images for: Immediate Mood Scaler: Tracking Symptoms of Depression and Anxiety Using a Novel Mobile Mood Scale
Source: JMIR Mhealth Uhealth. 2017 Apr 12;5(4):e44. doi: 10.2196/mhealth.6544 (PMC5406620; doi:10.2196/mhealth.6544)

#### Multimedia Appendix 4.

Parallel analysis based on 1,000 simulations of raw data.

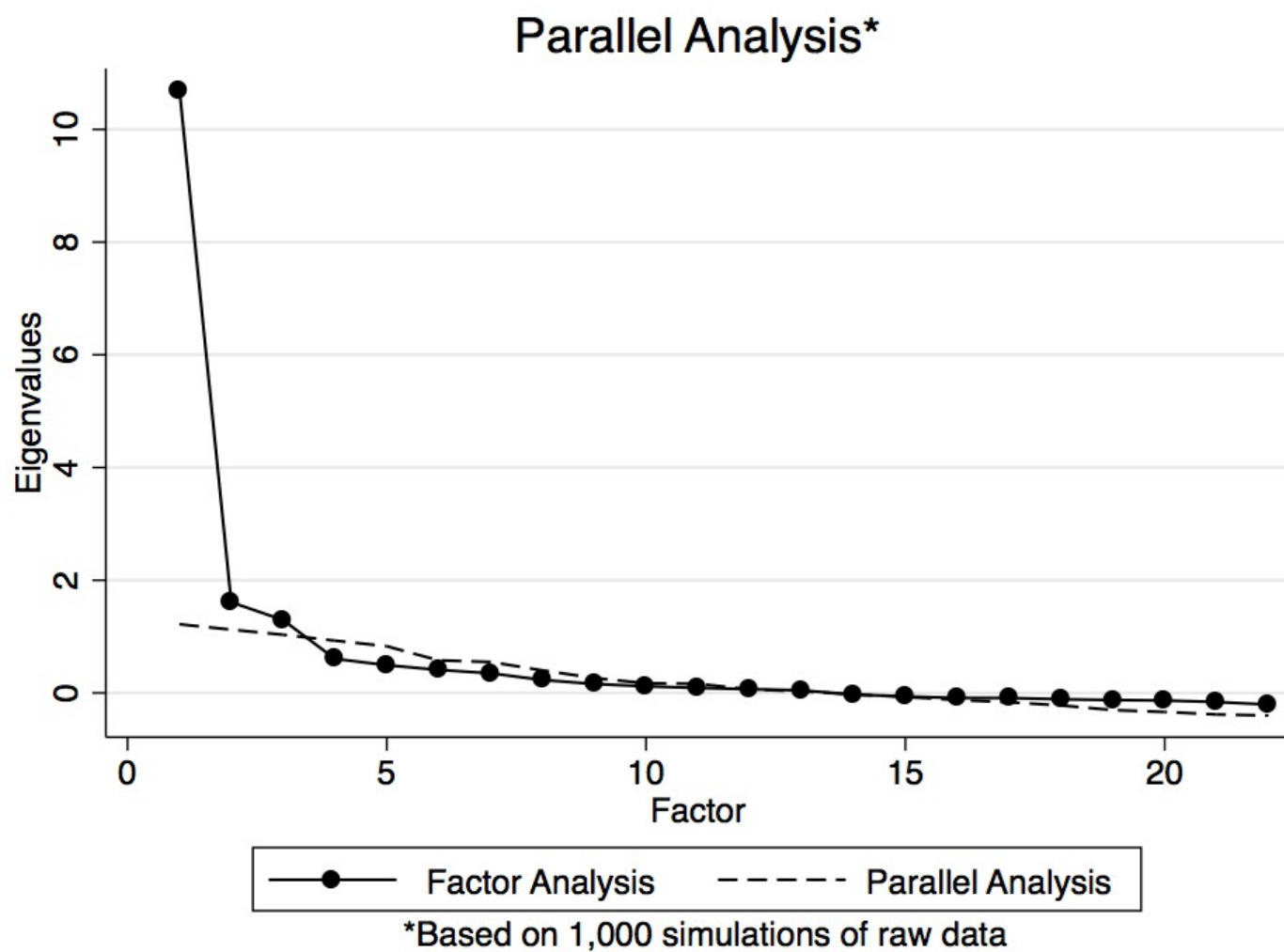

Supplement: Multimedia Appendix 4 [file mhealth_v5i4e44_app4.pdf]
