# Supplementary material for: Immediate Mood Scaler: Tracking Symptoms of Depression and Anxiety Using a Novel Mobile Mood Scale
Source: JMIR Mhealth Uhealth. 2017 Apr 12;5(4):e44. doi: 10.2196/mhealth.6544 (PMC5406620; doi:10.2196/mhealth.6544)
Supplement: Multimedia Appendix 5 [file mhealth_v5i4e44_app5.pdf]

Multimedia Appendix 5.

Correlations (Pearson's r) between standardized measures and IMS

	PHQ-9	GAD-7	Rumination	IMS total	IMS-12	IMS12_depr	IMS12_anx
**PHQ-9**	1						
**GAD-7**	.78** (99)	1					
**Rumination**	.73** (65)	.73** (65)	1				
**IMS total**	.59** (110)	.57** (93)	.57** (64)	1			
**IMS-12**	.59** (110)	.54** (93)	.59** (64)	.97** (110)	1		
**IMS12_depr**	.57** (110)	.46** (93)	.53** (64)	.88** (110)	.92** (110)	1	
**IMS12_anx**	.49** (110)	.51** (93)	.53** (64)	.87** (110)	.89** (110)	.64** (110)	1

Note: number of participants (n) appears in parenthesis; ***P*<.001 (two-tailed). Abbreviations: IMS12_depr: depression subscale of the 12-item IMS; IMS12_anx: anxiety subscale of the 12-item IMS.
