# Supplementary material for: Immediate Mood Scaler: Tracking Symptoms of Depression and Anxiety Using a Novel Mobile Mood Scale
Source: JMIR Mhealth Uhealth. 2017 Apr 12;5(4):e44. doi: 10.2196/mhealth.6544 (PMC5406620; doi:10.2196/mhealth.6544)
Supplement: Multimedia Appendix 6 [file mhealth_v5i4e44_app6.pdf]

Multimedia Appendix 6.

Model results using IMS-12 Depression and Anxiety subscales for Depression (PHQ-9; top table) and Anxiety (GAD-7; bottom table)

IMS-12 Depression (n=17)				IMS-12 Anxiety (n=17)			
**Model**	*β*	***t***	***P*[A]**	**Model**	*β*	***t***	***P***
**Time**	.17	.81	.46	**Time**	.12	-.38	.71
**+ PHQ-9 Baseline**	4.00	9.47	<.001	**+ GAD-7 Baseline**	3.81	10.24	<.001
**+ IMS-12 Depression**	.67	1.84	.073	**+ IMS-12 Anxiety**	.97	-2.44	.019

[A]P values indicate whether the model provides a significant additional proportional reduction in errors relative to the previous model.

IMS-12 Depression (n=17)				IMS-12 Anxiety (n=17)			
**Model**	*β*	***t***	***P*[&]**	**Model**	*β*	***t***	***P***
**Time**	-.01	-.04	.96	**Time**	-.03	-.12	.91
**+ PHQ-9 Baseline**	4.74	11.67	<.001	**+ GAD-7 Baseline**	4.49	10.64	<.001
**+ IMS-12 Depression**	-.61	-1.98	.059	**+ IMS-12 Anxiety**	-.85	-2.29	.027
